# Risk-Based Pre-Admission Screening for Carbapenem-Resistant Enterobacterales (CRE): A Patient-Level Observational Study in a High-Endemic European Setting

**DOI:** 10.3390/microorganisms14061262

**Published:** 2026-06-03

**Authors:** Salvatore Altavilla, Daniela Loconsole, Nicoletta Di Pietro, Rossella Memmola, Donato Sivo, Francesco Di Gennaro

**Affiliations:** 1 Azienda Ospedaliero-Universitaria Consorziale Policlinico di Bari, 70124 Bari, Italy; 2 Clinic of Infectious Diseases, Department of Precision and Regenerative Medicine and Ionian Area (DiMePRe-J), University of Bari “Aldo Moro”, 70121 Bari, Italy

**Keywords:** carbapenem-resistant Enterobacterales, CRE, carbapenemase-producing Enterobacterales, pre-admission screening, infection prevention, hospital epidemiology

## Abstract

Multidrug-resistant organisms, particularly carbapenem-resistant Enterobacterales (CRE), represent a major global health threat. In settings with endemic circulation of carbapenem-resistant organisms, early identification of colonised patients before hospital admission may play a critical role in limiting in-hospital spread and guiding infection prevention strategies. We conducted a retrospective monocentric observational study including all patients evaluated for hospital admission in 2025. Patients presenting predefined epidemiological or clinical risk factors underwent risk-based pre-admission screening for CRE. Patient-level deduplication was applied to microbiologically positive records. Among 2694 patients evaluated for hospital admission, 1084 met predefined screening criteria and underwent rectal swab testing. Overall, 191 unique patients were confirmed as carriers of carbapenemase-producing Enterobacterales, corresponding to 17.6% of screened patients and 7.1% of the overall cohort evaluated for admission. KPC was the most prevalent carbapenemase gene (102/191, 53.4%), followed by NDM (57/191, 29.8%) and KPC/NDM co-production (14/191, 7.3%). Less frequent gene profiles included VIM, OXA-48, and combined carbapenemase patterns. In high-endemic healthcare settings, risk-based pre-admission screening may represent a pragmatic component of infection prevention pathways by supporting early identification of patients with probable CRE/CPE carriage. When analysed at the patient level, such programmes can provide useful operational and epidemiological information for admission management and infection control planning.

## 1. Introduction

Antimicrobial resistance (AMR) remains one of the major global public health threats, with substantial consequences for morbidity, mortality, healthcare expenditure, and the sustainability of hospital systems [1,2]. Among multidrug-resistant organisms, carbapenem-resistant Enterobacterales (CRE) are of particular concern because they are associated with limited therapeutic options, severe healthcare-associated infections, and the capacity for silent dissemination through intestinal colonisation [3,4,5].

Carbapenemase-producing Enterobacterales (CPE) represent a clinically and epidemiologically relevant subgroup of CRE. Carbapenemase genes may be carried by mobile genetic elements and can spread across bacterial species and healthcare settings. Invasive CRE/CPE infections, particularly those caused by *Klebsiella pneumoniae*, are frequently associated with poor outcomes, especially in patients with severe comorbidities or repeated healthcare exposure [6,7,8,9,10].

Europe shows marked geographical heterogeneity in CRE/CPE burden. Italy, and particularly Southern Italy, is recognised as a high-endemic context, with national and European surveillance documenting sustained circulation of carbapenem-resistant Klebsiella pneumoniae and carbapenemase-producing Enterobacterales, predominantly KPC-producing strains, with increasing attention to NDM and other carbapenemases [11,12,13,14,15].

Early identification of colonised patients is therefore a central component of infection prevention and control (IPC) programmes in endemic hospitals. Universal admission screening may be difficult to sustain because of laboratory workload, costs, bed availability, and organisational constraints. For this reason, many hospitals use risk-based strategies that target patients with predefined epidemiological or clinical risk factors [16,17,18,19,20].

However, the operational yield of risk-based pre-admission screening remains insufficiently characterised in real-world hospital-wide pathways. In addition, several studies report screening results at the sample or episode level, whereas patient-level analyses are more useful for admission management, isolation policies, cohorting decisions, and infection-control planning [21].

This study was conducted in a large tertiary-care referral hospital in Bari, Apulia Region, Southern Italy, a high-endemic area within the Italian and European CRE/CPE epidemiological context. The aim was to evaluate the yield of a risk-based pre-admission CRE screening programme by describing the demographic profile of screened patients, the molecular characterisation of detected carbapenemase genes, and the distribution of screening indications used to trigger rectal swab testing.

## 2. Materials and Methods

### 2.1. Study Design and Setting

This retrospective monocentric hospital-based observational study was conducted at the Azienda Ospedaliero-Universitaria Consorziale Policlinico di Bari, Bari, Apulia Region, Southern Italy. The hospital operates as a large tertiary-care referral centre for patients requiring complex medical and surgical care and is located in an Italian region where CRE/CPE circulation is considered endemic according to national and European surveillance data [11,14,15].

### 2.2. Study Population

**Figure 1:**
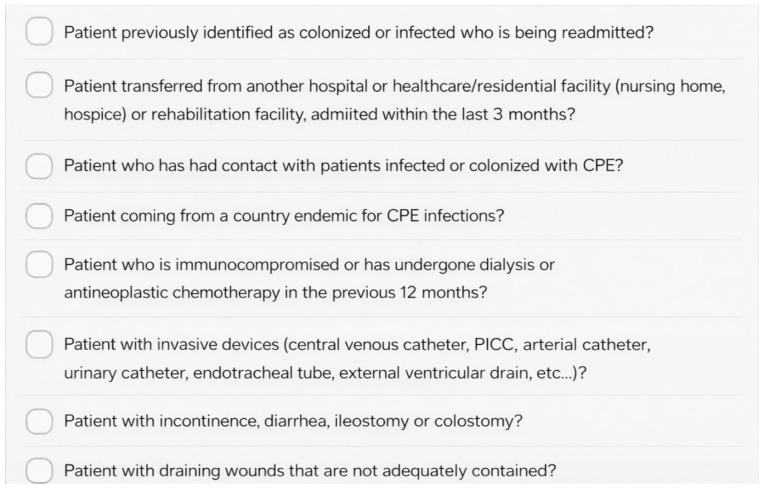
Structured risk-assessment checklist used during pre-admission evaluation to identify patients requiring rectal swab screening for carbapenem-resistant Enterobacterales (CRE). PICC: peripherally inserted central catheter.

All patients evaluated for hospital admission between 1 January and 31 December 2025 were considered for inclusion. During the study period, each patient underwent an infectious risk assessment before admission. According to the institutional protocol, CRE rectal swab screening was performed only when at least one predefined epidemiological or clinical risk criterion was identified using a structured checklist (Figure 1).

### 2.3. Screening Criteria and Data Management

Screening eligibility was determined using the institutional checklist shown in Figure 1. For analytical purposes, checklist items were grouped into predefined risk domains consistent with routine clinical practice: recent hospitalisation or transfer from a healthcare/residential facility; immunosuppression, dialysis, or recent antineoplastic chemotherapy; presence of invasive or indwelling medical devices; faecal or urinary incontinence or enterostomy; draining wounds or chronic skin lesions; and known contact with a CPE-positive patient. Screening could also be triggered by previous documented CRE/CPE colonisation or infection, origin from a country with endemic CPE circulation, or planned admission to selected high-risk hospital units according to local infection prevention procedures.

Recent hospitalisation was defined as any admission to an acute-care, rehabilitation, hospice, long-term care, or residential healthcare facility within the previous 3 months. Immunosuppression included active haematological or solid malignancy under treatment, ongoing chemotherapy, dialysis, long-term corticosteroid therapy, or other immunosuppressive treatments. Indwelling devices included urinary catheters, central venous catheters, peripherally inserted central catheters, tracheostomy or endotracheal tubes, feeding tubes, external ventricular drains, or comparable invasive devices present at evaluation.

Faecal or urinary incontinence was defined as documented loss of bowel or bladder control requiring containment devices or assistance in daily care. Draining wounds or chronic skin lesions included actively secreting wounds, pressure ulcers, surgical wounds, or chronic skin lesions with exudate. Known contact with a CPE-positive patient was defined as documented exposure to a patient known to be colonised or infected with carbapenemase-producing Enterobacterales during a previous hospitalisation or in another healthcare facility.

Patients could meet more than one screening criterion. When multiple screening records referred to the same patient during the study period, patient-level deduplication was applied and only the first screening episode was retained for the analytical dataset.

### 2.4. Sample Collection and Microbiological Methods

Rectal swab screening was used as the standard approach for the detection of CRE/CPE carriage in patients considered at risk. Rectal swabs were collected during the pre-admission infectious risk assessment by trained healthcare personnel and processed by the hospital microbiology laboratory according to routine standard operating procedures.

Samples were inoculated onto conventional and/or selective culture media suitable for the recovery of Gram-negative Enterobacterales, including selective or chromogenic media for the presumptive detection of carbapenem-resistant organisms, according to the laboratory diagnostic workflow and the manufacturer’s instructions. Presumptive resistant colonies were further processed for species-level identification using validated automated laboratory systems, including MALDI-TOF mass spectrometry (VITEK MS, bioMérieux, Marcy-l’Étoile, France) and/or automated biochemical identification platforms. Antimicrobial susceptibility testing was performed using automated systems and, when required, complemented by disc diffusion, gradient diffusion, or broth microdilution methods. Results were interpreted according to EUCAST clinical breakpoints applicable during the study period.

When molecular confirmation was clinically indicated, PCR-based assays were performed using validated commercial real-time PCR or multiplex PCR platforms. Molecular testing targeted the main carbapenemase genes relevant to the study, including blaKPC, blaNDM, blaVIM, blaIMP, and blaOXA-48-like. Commercial molecular assays were performed according to the manufacturers’ instructions and included routine internal quality-control procedures, with positive and negative controls according to laboratory practice.

As this was a retrospective observational study based on routinely generated diagnostic reports, the analysis did not involve the development, comparison, or independent analytical validation of a new molecular assay. Molecular results were extracted from validated laboratory reports generated within the routine diagnostic workflow. Therefore, primer sequences, complete amplification conditions, and proprietary assay specifications were not available in the research dataset and could not be reported in full. In this study, CRE refers to carbapenem-resistant Enterobacterales, whereas CPE denotes isolates carrying carbapenemase genes confirmed by molecular testing.

### 2.5. Statistical Analysis

Categorical variables are reported as counts and percentages, and continuous variables as medians with interquartile ranges (IQR). Screening rates were calculated using the overall cohort evaluated for admission as the denominator, whereas positivity rates were calculated using the number of screened patients as the main denominator, unless otherwise specified. Because screening indications were non-mutually exclusive and microbiologically positive records were analysed after patient-level deduplication, the final analysis was descriptive. No multivariable model was presented, as the available retrospective dataset did not allow stable and fully reproducible patient-level modelling of independent associations between individual screening criteria and CPE positivity.

### 2.6. Ethical Considerations

The study was retrospective and observational, used anonymised routinely collected clinical, administrative, and microbiological data generated during standard care and infection prevention activities, and did not involve any intervention or deviation from routine practice. Ethical review and informed consent were therefore waived in accordance with institutional procedures, Italian legislation, and the EU General Data Protection Regulation (Regulation EU 2016/679 [22]).

## 3. Results

During the study period, 2694 patients were evaluated for hospital admission and underwent infectious risk assessment. Among them, 1084 patients met predefined screening criteria and underwent rectal swab testing for CRE colonisation, corresponding to 40.2% of the overall cohort evaluated for admission. Demographic characteristics of the study population are reported in Table 1.

The cohort was predominantly elderly, with a median age of 73 years (IQR 59–82), and showed a slight male predominance (54.4%).

Indications for screening are summarised in Table 2. Screening could be triggered either by at least one clinical history-based CRE risk criterion included in the institutional checklist or by planned admission to selected high-risk hospital units requiring pre-admission rectal swab screening according to local infection prevention procedures. Patients could present more than one screening indication; therefore, counts refer to screening indications and not to mutually exclusive patient groups.

Overall, 1128 screening indications were recorded among the 1084 screened patients. Most patients had one screening indication (1043/1084, 96.2%), whereas 38 (3.5%) had two indications and 3 (0.3%) had three indications. Percentages in Table 2 are calculated using the 1084 screened patients as the denominator. Recent hospitalisation or recent admission from another healthcare/residential facility was the most frequent indication (510/1084, 47.0%), followed by immunosuppression/dialysis/chemotherapy (182/1084, 16.8%), faecal or urinary incontinence/diarrhoea/ostomy (133/1084, 12.3%), invasive devices (102/1084, 9.4%), planned admission to a high-risk hospital unit (102/1084, 9.4%), draining wounds (91/1084, 8.4%), previous CRE/CPE colonisation or infection (5/1084, 0.5%), and known contact with a CPE-positive patient (3/1084, 0.3%).

Overall, 191 unique patients were found to carry carbapenemase-producing Enterobacterales after patient-level deduplication of microbiologically positive records, corresponding to a positivity rate of 17.6% among screened individuals and 7.1% of the overall cohort evaluated for admission. The molecular characterisation of carbapenemase genes among CPE-positive patients is reported in Table 3.

Because patients without screening indications did not routinely undergo rectal swab testing, the colonisation rate among the 1610 non-screened individuals could not be directly estimated from the available retrospective data. Consequently, the observed 7.1% positivity rate in the overall admission cohort should be interpreted as a minimum detected burden rather than as the true prevalence of CRE/CPE colonisation among all patients evaluated for admission. In operational terms, the programme detected CPE carriage among patients enriched for epidemiological, clinical, or organisational risk, while the potential proportion of colonised individuals missed among those without screening indications remains unknown.

Because the molecularly positive records were consolidated through patient-level deduplication and screening indications were non-mutually exclusive, inferential estimates for individual criteria were not retained in the final revised analysis. The screening pathway is therefore presented descriptively, focusing on the yield of the programme and on the molecular distribution of carbapenemase genes among positive patients.

KPC and NDM accounted for 83.2% of detected carbapenemase genes, indicating a local CPE pattern dominated by KPC, with a substantial contribution of NDM.

## 4. Discussion

### 4.1. Principal Findings and Comparison with Published Evidence

This study provides real-world patient-level data on the operational yield of a risk-based pre-admission CRE screening strategy in a high-endemic Southern Italian healthcare setting. The programme identified 191 CPE carriers among 1084 screened patients, corresponding to a positivity rate of 17.6% among screened individuals and 7.1% of the overall cohort evaluated for admission. This yield is clinically relevant because the screened population was not selected on the basis of culture positivity but on predefined epidemiological and clinical criteria applied before admission. In this sense, the findings support the practical value of targeted screening in endemic hospitals, where universal screening may be difficult to sustain and where early recognition of colonised patients can guide isolation, cohorting, and bed allocation decisions [14,16,18,19].

A direct estimate of colonisation among non-screened patients would require a different study design. In the present pathway, absence of a screening indication generally meant that rectal swab testing was not performed; therefore, the negative group cannot be interpreted as microbiologically negative. This distinction is important for interpretation of the screening yield. The programme can demonstrate how many carriers were detected among patients selected by predefined criteria, but it cannot, by itself, quantify the sensitivity of the algorithm or the proportion of total colonisation captured by the risk-based approach. For this reason, the overall cohort positivity rate should be considered a conservative operational measure of detected burden rather than a prevalence estimate. A prospective validation phase, in which a representative sample of patients without risk criteria is also screened, would be needed to estimate missed colonisation and to calculate negative predictive value.

The resource implications of such a validation strategy are substantial. Universal admission screening, or even systematic sampling of non-screened patients, would increase the number of rectal swabs, microbiology laboratory workload, consumable use, personnel time, reporting activity, and infection-prevention follow-up. In hospitals with endemic CRE/CPE circulation, additional positive findings may also increase demand for single rooms, cohort areas, contact precautions, environmental cleaning, prevention of environmental contamination, and bed-management coordination [18,23,24]. These costs must be balanced against the potential benefit of identifying otherwise unrecognised carriers before ward admission. The present findings therefore support a pragmatic interpretation: risk-based screening is operationally feasible and has a high observed yield among selected patients, but future implementation research should compare its performance and resource consumption with universal or hybrid screening strategies.

From a practical perspective, the choice between universal screening, targeted screening, and hybrid validation should be guided by measurable operational indicators rather than by microbiological yield alone. Relevant indicators include the number of swabs required to detect one additional carrier, time from admission evaluation to microbiological result, proportion of positive patients isolated before ward placement, availability of isolation beds, frequency of delayed admissions related to infection-control constraints, and downstream impact on patient flow. These indicators are particularly important in tertiary hospitals, where patients with complex comorbidities may require urgent placement in specialised wards and where isolation capacity is often limited. A risk-based strategy may therefore remain preferable when resources are constrained, provided that the criteria are periodically audited and updated according to local epidemiology. Conversely, if a prospective audit shows substantial missed colonisation among patients without predefined criteria, expansion of screening indications or periodic universal point-prevalence screening may be justified. This operational evaluation would allow hospitals to move from a purely descriptive screening policy to a learning infection-prevention system in which epidemiological data, laboratory capacity, and bed-management decisions are continuously aligned.

The positivity rate observed in this cohort should be interpreted in relation to the epidemiological setting and to the risk-enriched nature of the screened population. Italy remains one of the European countries with a substantial burden of carbapenem-resistant Klebsiella pneumoniae and carbapenemase-producing Enterobacterales, although important geographical and institutional differences persist [11,14,15,25]. Our results are therefore not intended to estimate the prevalence of CRE colonisation among all hospital admissions. Rather, they quantify the yield of a real-world risk-based pathway applied in a tertiary-care hospital located in an area with sustained CPE circulation. This distinction is important when comparing our findings with studies from lower-endemic countries or with surveillance studies based on bloodstream infections rather than colonisation screening.

Compared with reports from other European settings, the present cohort shows both expected and locally specific features. Studies and surveillance reports from endemic regions have repeatedly shown that previous healthcare exposure, transfer from healthcare facilities, severe underlying disease, and the presence of invasive devices increase the likelihood of CRE/CPE carriage [14,16,19,22,26,27]. In the present study, the screening pathway was intentionally designed to capture this risk-enriched population before admission rather than to estimate the independent predictive weight of each individual criterion. Therefore, the criteria should be interpreted as operational triggers for infection prevention and bed-allocation decisions, not as mutually exclusive causal risk factors.

### 4.2. Interpretation of the Risk-Based Screening Strategy

The main practical implication of these findings is that risk-based pre-admission screening can function as an operational tool rather than as a purely microbiological test. In high-endemic settings, the clinical problem is not only whether CPE can be detected, but whether detection occurs early enough to influence the admission pathway. A positive result before or at the time of admission may affect room placement, contact precautions, cohort isolation, nursing workload, and communication between the emergency department, admitting wards, infection prevention staff, and bed management teams. This organisational dimension is particularly relevant when isolation capacity is limited and when delayed identification may lead to avoidable exposure of other patients [18,24,28].

At the same time, a risk-based strategy inevitably creates a selective dataset. Because only patients meeting predefined criteria were tested, the study cannot determine how many colonised patients without recognised risk factors were missed. This is a known trade-off of targeted screening: it improves feasibility and concentrates laboratory resources on higher-risk patients, but it may reduce sensitivity at the population level. For this reason, local epidemiology, periodic reassessment of screening criteria, and feedback from infection prevention surveillance are essential. In institutions where NDM, OXA-48-like, or mixed carbapenemase patterns are increasing, risk algorithms may need to be revised to include additional exposures such as recent admission abroad, repeated interfacility transfers, or previous colonisation documented in electronic records [14,20,25,29].

### 4.3. Carbapenemase Gene Distribution and Epidemiological Interpretation

The molecular characterisation of carbapenemase genes showed a pattern dominated by KPC, followed by NDM and KPC/NDM co-production, with smaller numbers of VIM, OXA-48, and combined carbapenemase profiles. The predominance of KPC is coherent with the Italian epidemiological background, where KPC-producing Klebsiella pneumoniae has historically represented a major driver of CRE endemicity. However, the substantial proportion of NDM-positive patients also indicates a more heterogeneous resistance landscape, consistent with recent European reports documenting the increasing circulation of metallo-beta-lactamases and the diversification of carbapenemase mechanisms in healthcare settings.

The identification of KPC/NDM co-producers deserves specific attention. Although they represented a smaller proportion of positive patients than single-gene KPC or NDM detections, co-production of carbapenemases may indicate the accumulation of resistance determinants in strains or plasmids circulating within highly exposed healthcare populations. From an infection prevention perspective, co-producers are relevant because they may be associated with complex transmission networks and because their detection can complicate both laboratory interpretation and therapeutic decision-making. Their presence in a pre-admission screening cohort suggests that molecular confirmation should not be limited to the detection of a single expected carbapenemase type, especially in endemic settings where multiple mechanisms may coexist [14,20,29].

After correction of the gene dataset, the single record previously labelled as NIV was reviewed as a typographical error and classified as VIM. This correction does not change the overall number of CPE-positive patients but slightly modifies the distribution of less frequent carbapenemase profiles. The presence of VIM and OXA-48-associated profiles, although less common than KPC and NDM, remains epidemiologically relevant because these enzymes may be associated with different diagnostic, therapeutic, and transmission-control challenges. Their detection supports the need for molecular confirmation rather than reliance on phenotypic carbapenem resistance alone.

VIM, OXA-48, and mixed carbapenemase profiles were less frequent than KPC and NDM but remain epidemiologically meaningful. OXA-48-like carbapenemases may be difficult to detect phenotypically in some contexts because carbapenem minimum inhibitory concentrations can be variable, whereas VIM and NDM belong to the metallo-beta-lactamase group and may limit therapeutic options. The presence of co-produced carbapenemases, including KPC/NDM and other combined profiles, is particularly relevant because it may reflect cumulative healthcare exposure, repeated antimicrobial selection pressure, and complex transmission networks in endemic hospital environments.

Overall, the carbapenemase distribution observed in this study has two implications. First, local endemicity appears to remain strongly shaped by KPC, in line with the Italian historical pattern. Second, the coexistence of NDM, KPC/NDM co-production, OXA-48, VIM, and other combined profiles indicates that local screening programmes should not be designed around a single resistance mechanism. In practical terms, this supports the use of screening approaches able to identify multiple carbapenemase families and reinforces the need for close collaboration between microbiology laboratories, infection prevention teams, and admission management pathways.

### 4.4. Clinical, Microbiological, and Organisational Implications

From a clinical perspective, the early identification of CPE carriers may help clinicians recognise patients at increased risk of subsequent difficult-to-treat infections, particularly when severe illness, invasive devices, immunosuppression, or recent healthcare exposure are present. Although colonisation does not necessarily imply infection, it can inform empirical therapy in selected high-risk scenarios and can support antimicrobial stewardship by avoiding both undertreatment and unnecessary escalation. These implications are especially important in settings where KPC and metallo-beta-lactamases coexist, because the resistance mechanism may influence the expected activity of newer antimicrobial combinations [9,20,29,30]. These considerations are also relevant in surgical pathways, where colonisation by multidrug-resistant Gram-negative bacteria may influence perioperative infection-prevention strategies [31].

From a microbiological perspective, the findings reinforce the value of combining phenotypic detection with molecular confirmation. Culture-based screening identifies colonised patients and supports infection prevention actions, whereas molecular characterisation clarifies the underlying carbapenemase mechanism. In routine practice, this distinction is important because CRE is a phenotypic category, while CPE indicates carbapenemase production confirmed by molecular testing. The present study deliberately reports CPE-positive patients after validation of matched records, thereby reducing the risk of overestimating the burden through duplicated samples or unmatched laboratory entries.

From an organisational perspective, screening results should be integrated into patient-flow management. A positive screening result is actionable only if it triggers timely isolation, clear communication, appropriate documentation in the electronic record, and coordination between clinical teams and infection prevention personnel. In this respect, pre-admission screening can be considered a system-level intervention that links microbiology, hospital logistics, and clinical care. This interpretation is consistent with the growing recognition that CRE control in endemic hospitals requires multimodal strategies rather than isolated laboratory testing [14,16,18,24,28].

### 4.5. Methodological Considerations Regarding Molecular Detection

The reviewer correctly highlights that detailed reporting of molecular methods improves reproducibility. In the present study, however, PCR testing was performed as part of routine diagnostic activity and the research dataset contained validated laboratory results rather than raw molecular run files. The study was not designed to develop, compare, or analytically validate PCR assays. Therefore, primer sequences, amplification conditions, and full assay-validation parameters were not available for independent reporting. To improve transparency, the Methods section now specifies that molecular results were extracted from validated routine laboratory reports and that PCR was used to detect the main carbapenemase genes reported in the Results. This limitation should be considered when interpreting the molecular component of the study.

This methodological constraint does not invalidate the epidemiological interpretation of the screening pathway, but it narrows the level of laboratory reproducibility that can be claimed. The reliability of the molecular results depends on the quality-assured routine workflow of the hospital microbiology laboratory rather than on a study-specific molecular protocol. Future prospective studies should collect and report the complete molecular platform information, including commercial assay name, manufacturer, target genes, internal controls, positive and negative controls, amplification conditions, interpretation criteria, and external quality-assessment participation. Such reporting would allow a more complete assessment of analytical validity and inter-laboratory comparability.

### 4.6. Limitations

This study has several limitations. First, the analysis reflects the experience of a single tertiary-care hospital located in a high-endemic setting, which may limit the generalisability of the findings to other healthcare contexts with different epidemiological patterns. Second, screening was restricted to patients meeting predefined risk criteria; therefore, the results do not provide an estimate of CRE colonisation prevalence among the overall population of hospital admissions and cannot evaluate the sensitivity of the screening checklist among patients without recognised risk factors.

Third, the retrospective structure of the available data and the non-mutually exclusive nature of screening indications limited the possibility of presenting stable and fully reproducible patient-level risk-factor models. For this reason, the final analysis was kept descriptive rather than inferential. This limitation reduces the ability to estimate the independent contribution of each screening criterion to CPE positivity, but it avoids overinterpretation of unstable odds ratios generated from an enriched and operationally selected screening population.

Fourth, the study relied on routinely collected retrospective clinical, administrative, and microbiological data. Although patient-level deduplication was applied and unmatched records were excluded, residual misclassification, incomplete documentation of screening criteria, and data-entry inaccuracies cannot be entirely ruled out. Finally, detailed laboratory platform information, including primer sequences and amplification conditions, was not available in the research dataset because PCR testing was performed within routine diagnostic workflows. This limitation restricts the reproducibility of the molecular methods, although the reported results remain useful for describing the operational yield and gene distribution observed in routine pre-admission screening.

## 5. Conclusions

In high-endemic healthcare settings, risk-based pre-admission screening may represent a pragmatic and operationally feasible component of infection prevention pathways. By targeting patients with predefined epidemiological and clinical risk factors, this approach enables early identification of individuals with probable CRE/CPE carriage before hospital admission, facilitating timely implementation of contact precautions, optimised bed allocation, and cohorting strategies.

When analysed at the patient level, such programmes provide meaningful epidemiological insights into the distribution of carbapenemase-producing Enterobacterales within high-risk populations and help refine screening policies based on real-world data. Although this study was not designed to estimate the independent predictive value of each screening criterion, it shows that a structured risk-based pathway can identify a clinically relevant burden of CPE carriage before admission.

Overall, risk-based screening should be interpreted not only as a diagnostic intervention but also as a system-level organisational tool that can support infection control planning, resource allocation, and patient flow management in healthcare systems with endemic CRE circulation. Future studies should evaluate the integration of such screening strategies with broader organisational and antimicrobial stewardship interventions to further optimise their impact.

## Figures and Tables

**Table 1:** Demographic characteristics of the overall study cohort.

Variable	Overall Cohort (*n* = 2694)
Male sex, *n* (%)	1465 (54.4%)
Female sex, *n* (%)	1229 (45.6%)
Median age, years (IQR)	73 (59–82)

**Table 2:** Indications for CRE screening among eligible patients (*n* = 1084).

Screening Indication	*n*	%
Recent hospitalisation or admission from another hospital, healthcare/residential facility, or rehabilitation facility within the previous 3 months	510	47.0
Immunosuppression, dialysis, or antineoplastic chemotherapy within the previous 12 months	182	16.8
Faecal or urinary incontinence, diarrhoea, ileostomy, or colostomy	133	12.3
Presence of invasive devices, including central venous catheter, peripherally inserted central catheter (PICC), arterial catheter, urinary catheter, endotracheal tube, or external ventricular drain	102	9.4
Planned admission to a high-risk hospital unit requiring pre-admission CRE screening	102	9.4
Draining wounds not adequately contained	91	8.4
Previously identified as colonised or infected with CRE/CPE and readmitted	5	0.5
Contact with patients colonised or infected with CPE	3	0.3
Coming from a country endemic for CPE infections	0	0.0

**Table 3:** Carbapenemase genes detected among deduplicated CPE-positive patients (*n* = 191).

Carbapenemase Gene	*n*	%
KPC	102	53.4
NDM	57	29.8
KPC/NDM	14	7.3
VIM	8	4.2
OXA-48	3	1.6
KPC/OXA-48	3	1.6
NDM/OXA-48	2	1.0
KPC/NDM/OXA-48	1	0.5
KPC/NDM/VIM	1	0.5

Note: Percentages are calculated over all deduplicated CPE-positive patients (*n* = 191).

## Data Availability

The data presented in this study are available upon reasonable request from the corresponding authors.
